# Interaction between long noncoding RNA and microRNA in lung inflammatory diseases

**DOI:** 10.1002/iid3.1129

**Published:** 2024-01-10

**Authors:** Jiaqi Li, Shengyu Huang, Liangliang Shi, Guochang Chen, Xiaoxiao Liu, Mingzhuo Liu, Guanghua Guo

**Affiliations:** ^1^ Medical Center of Burn Plastic and Wound Repair The First Affiliated Hospital of Nanchang University Nanchang China

**Keywords:** lncRNA, lung inflammatory diseases, miRNA, RNA interaction

## Abstract

**Background:**

Non‐coding RNAs (ncRNAs) are a group of RNAs that cannot synthesize proteins, but are critical in gene expression regulation. Long non‐coding RNAs (lncRNAs) and microRNAs (miRNAs), the two major family members, are intimately involved in controlling immune response, cell proliferation, apoptosis, differentiation and polarization, and cytokine secretion. Their interactions significantly influence lung inflammatory diseases and could be potential therapeutic targets.

**Objectives:**

The review aims to elucidate the role of ncRNAs, especially the interactions between lncRNA and miRNA in lung diseases, including acute and chronic lung inflammatory diseases, as well as lung cancer. And provide novel insights into disease mechanisms and potential therapeutic methods.

**Methods:**

We conducted a comprehensive review of the latest studies on lncRNA and miRNA in lung inflammatory diseases. Our research involved searching through electronic databases like PubMed, Web of Science, and Scopus.

**Results:**

We explain the fundamental characteristics and functions of miRNA and lncRNA, their potential interaction mechanisms, and summarize the newly explorations on the role of lncRNA and miRNA interactions in lung inflammatory diseases.

**Conclusions:**

Numerous lncRNAs and miRNAs have been found to partipicate in all stages of lung inflammatory diseases. While ncRNA‐based therapies have been validated and developed, there remain challenges in developing more stable and effective drugs for clinical use.

## Graphical abstract

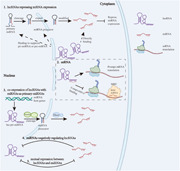

## 1 INTRODUCTION

Noncoding RNAs (ncRNAs), were long ignored as “noise” due to their inability to encode proteins, have recently gained notice for the critical function in gene transcription and translation. Only about 2% of human genes have protein‐coding potential, the remaining 98% known as noncoding RNAs, mainly includes ribosomal RNA (rRNA), transfer RNA (tRNA), ribozymes, small nuclear RNA (snRNA), small nucleolar RNA (snoRNA), microRNAs (miRNAs), long noncoding RNAs (lncRNAs), and circular RNA (circRNAs).^1^ The newly technologies such as high‐throughput sequencing (HTS) made it possible to detect RNA's structure and expression, biological functions, and interactions of RNA‐RNA, RNA‐DNA, and RNA‐protein with high sensitivity and accuracy. This has opened new insights of their functions, molecular mechanisms, and relationship with diseases across multiple species.^2^

LncRNA and miRNA bind and interact with each other in variety ways, influencing downstream gene expression. Increasing evidence shows that RNA‐RNA interactions, especially lncRNA‐miRNA interactions, are crucial for gene expression in physiological and pathological processes. Furthermore, lncRNA and miRNA, which are expressed in all kinds of diseases, involved in gene regulation and cellular metabolic process, may be ideal biomarkers or therapeutic targets for cancer.^3^

LncRNA‐miRNA interactions influence cell proliferation, apoptosis, differentiation, polarization, and cytokine secretion. This review aims to provide a comprehensive overview of several potential regulatory mechanisms of lncRNA‐miRNA interactions, including lncRNAs repressing miRNA expression, competitive endogenous RNA (ceRNA), co‐expression of lncRNAs with miRNAs as primary‐miRNAs, miRNAs negatively regulating lncRNAs, and mutual repression between lncRNAs and miRNAs. Based on these mechanisms, the latest studies are reviewed to reveal how lncRNA‐miRNA interactions in relation to several prevlant acute and chronic lung inflammatory diseases, as well as lung cancer.

## 2 LncRNA‐miRNA INTERACTIONS

### 2.1 LncRNA

LncRNAs, longer than 200 nt, stimulate or inhibit transcription at the transcriptional level, influence mRNA splicing, editing, translation, or stability at the posttranscriptional level, and performing epigenetic regulation.^2^ As reside in various cells and subcellular localizations, different lncRNAs serve diverse activities at different times. The following categories can be determined by where they are located on coding genes: (1) Intergenic (has no overlap with protein‐coding genes); (2) Antisense (enriched around the promoter or terminator ends of the sense transcript); (3) Intronic (located in the area of gene coding sites); (4) Divergent lncRNA (abundanted in the vicinity of transcription start sites); (5) Pseudogenes (genes that have no potential of coding).^4^ Divergent lncRNAs, which are head‐to‐head overlap with the coding genes, account for about 20% of all lncRNAs. Divergent lncRNAs are strongly tied to essential growth and developmental regulatory genes. Their functions are associated with those of their neighboring coding genes, which can regulate those genes in cis and encourage the diversification of higher eukaryotic phenotypes.^5^ With a countless number of lncRNA, more are continually being found and labeled, and many lncRNAs' roles have not yet been thoroughly investigated.

### 2.2 MicroRNA

MicroRNAs are small endogenous noncoding RNAs that are 18–25 nucleotides in size, with high conservation and specificity.^6^ MiRNA are crucial regulators of gene expression as posttranscriptional silencing by binding and inhibiting target genes' translation, widely involved in growth development and pathological processes.^7^

Intergenic miRNAs be transcribed with their own promoter independently, whereas intragenic miRNAs be transcribed together with the host gene. Long chain primary transcripts (pri‐miRNAs) be transcribed and then cuts by Drosha protein complex and generates pre‐miRNA, after transported to cytoplasm from nucleus, pre‐miRNA being cut and modified by Dicer enzymes to form mature miRNAs.^8^ By partially complementary binding to the target gene's 3′ noncoding region (3′UTR), miRISCs (miRNA‐induced silencing complexes) leading to transcriptional repression of the target mRNA with no impact on mRNA stability. Completely complementary binding directly cleaves target mRNA. Besides, miRNA also could lead mRNA deadenylation, transcriptional repression or cleavage may trigger the deadenylative processes of mRNA.^9^ It has been also reported that miRNAs binding to the 5′‐UTR of mRNA may activate translation.^10^ MiRNAs suppression and activation of target mMiRNA regulate nearly all biological functions, including cell division, proliferation, differentiation, apoptosis, and cell cycle.^11^

MiRNA involved in genes regulation networks, miR‐21, miR‐29 family, miR‐27(a/b), miR‐34 in oral fluids were shown to be biomarkers in the tooth movement, modulating the process of osteoblastogenesis, osteoclastogenesis, and extra‐cellular matrix conformation posttranscriptionally, and regulating the physiological processes of orthodontic‐related bone and tissue remodeling.^12^ MiR‐1 and miR‐133, act as co‐transcriptional and co‐regulatory factors,^13^ that are highly expressed in injured myocardial tissue, encapsulated in exosomes and released into circulation, mediating the mobilization of bone marrow progenitor cells from bone marrow to the peripheral circulation and participating in the repair of myocardial ischemia.^14^

### 2.3 The potential mechanisms of lncRNA‐miRNA interaction

#### 2.3.1 LncRNA repressing miRNA expression

LncRNA bind with miRNA and suppress its expression by common binding sites, or lncRNA directly suppressing miRNA precursors or primary miRNAs, thereby lowering transcription product creation. Lnc‐PFAR stems pre‐miR‐141 maturation by binding with pre‐miR‐141 in 72 Nucleotide binding domain, thus reducing miR‐141 expression.^15^ Rather than direct bind and interact with mature miRNA, lnc uc.173 combined with pri‐miR‐195 in its central stem region, destabilizing and enhancing degradation of pri‐miR‐195, thus inhibiting Dorsha‐mediated pri‐miRNA processing to pre‐miRNA, and deregulate the expression of miR‐195.^16^ In addition, the binding of lncRNA to miRNA, or miRNA binding sponge, exerts a role in inhibiting miRNA function, while further affect the mRNA levels of miRNA downstream targets, and this mechanism of interaction by competitively binding miRNA to mRNA is known as the ceRNA mechanism.

#### 2.3.2 Competitive endogenous RNA (CeRNA)

ceRNA reveals a novel role of RNA‐RNA interaction, has gained attention in recent years. LncRNA binding with the miRNA and blocking it from attaching to the miRNA response element (MRE) at the 3′‐UTR end, which allowed the translation of mRNA continue. LncRNA in this regulation also known as miRNA sponge, the ceRNA mechanism refers to the competitive binding between lncRNAs and mRNAs.^17^ Other RNAs, such as mRNA, pseudogenes, and circRNA also can serve in this way as ceRNA.^18^

LncRNA Sox2ot from exosomes derived from highly invasive tumor cells, binding to miR‐200 as ceRNA mechanism, upregulate Sox2, to induce epithelial‐mesenchymal transition (EMT) and stem cell like properties in different tumor cells, plays important roles in pancreatic ductal adenocarcinoma invasion and metastasis.^19^ Lnc SNHG1 promote neuroinflammation, and neuronal toxicity via different mechanisms, in the process of Alzheimer's disease, it could act as ceRNA for miR‐137, targeting KREMEN1 in the human primary neuron (HPN) cells, reduce cell viability, promote cell apoptosis, decrease mitochondrial membrane potential, and the protein levels of cytochrome C.^20^

#### 2.3.3 Co‐expression of lncRNAs with miRNAs as primary‐miRNAs

Some miRNAs host genes can encode both lncRNA and miRNA, one of the functions of this class of lncRNAs is to act as primary miRNA, termed lnc‐pri‐miRNAs, able to produce miRNAs.^21^ Among them, lnc LOC646329 can act as both a pri‐miRNA to produce miR‐29a/b1, and a transcriptional enhancer to activate neighboring oncogenes and promote Glioblastoma cell proliferation.^22^ The genomic organization of lnc MIR100HG, is located on human chromosome 11 (hsa chr11), generate miR‐100 and miR‐125b, which co‐represses several Wnt/β‐catenin negative regulators, to rescue cetuximab responsiveness of cetuximab‐resistant colorectal cancer and head and neck squamous cell cancer cell lines.^23^

#### 2.3.4 miRNA negatively regulating lncRNA

TMPO antisense RNA 1 (TMPO‐AS1) gene, located on chromosome 12, served as the diagnostic and prognostic marker of lung adenocarcinoma (LUAD). MiR‐383‐5p binding with lnc TMPO‐AS1 and inhibits its expression, significantly reducing tumorigenesis and progression of LUAD.^24^

MALAT1 is each individually suppressed by MiR‐216a, miR‐216b, and miR‐217. Especially, miR‐216a and MALAT1's association further induced G2/M arrest and cell cycle inhibition, decreased cell viability and apoptosis in pancreatic cancer cells.^25^

#### 2.3.5 Mutual repression between lncRNAs and miRNAs

**Figure 1:**
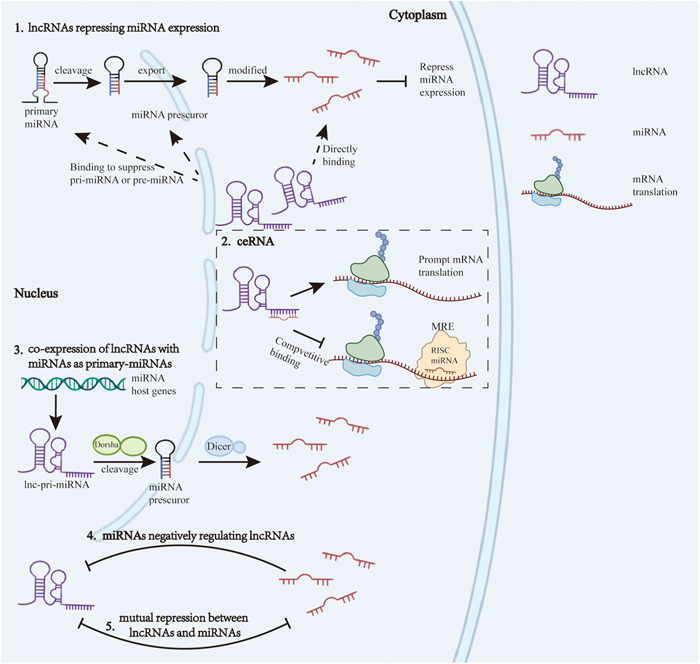
Five potential regulatory mechanisms of lncRNA‐miRNA interactions: (1) lncRNAs repressing miRNA expression; (2) ceRNA; (3) co‐expression of lncRNAs with miRNAs as primary‐miRNAs; (4) miRNAs negatively regulating lncRNAs; (5) mutual repression between lncRNAs and miRNAs.

While lnc MIR31HG can act as a sponge to bind miR‐193b, miR‐193b can also directly target two binding sites on lnc MIR31HG, negatively regulates lnc MIR31HG levels, induces apoptosis and G1/the S phase arrest, and reduces the cell growth of pancreatic ductal adenocarcinoma. These mutually inhibitory effects contribute to the growth of tumors.^26^ A negative feedback pathway is formed between lnc MALAT1 and miR‐200c‐3p. On the one hand, miR‐200c‐3p can mediate the silencing of MALAT1, which plays a significant role in the migration and invasion of pancreatic ductal adenocarcinoma and can be used as a prognostic indicator. On the one hand, the high expression of lnc MALAT1 in pancreatic ductal adenocarcinoma inhibits the expression of miR‐200c‐3p^27^ (Figure 1).

## 3 ACUTE LUNG INFLAMMATORY DISEASES

### 3.1 Pneumonia

Pneumonia can be brought on by a wide range of microorganisms, including bacteria, viruses, mycoplasma, and fungus. When microorganisms invade, nonspecific immunity responds positively with the release of inflammatory factors and other immunomodulators from macrophages, which are the primary source of inflammatory factors. In the same time, macrophages directly engulf and kill microorganisms. Following this, neutrophils and other immune cells are recruited, then neutrophils perform pathogen clearance by phagocytosis of lysosomes, formation of neutrophil extracellular traps (NETs), and degranulation to release myeloperoxidase (MPO), gelatines B (MMP9), while also producing inflammatory factors and chemokines.^28^ Surface active proteins (SP‐A, SP‐D, etc.) synthesized by alveolar II epithelial cells directly inhibit microbial activity.

#### 3.1.1 Bacterial infections

The most common pathogens of hospital‐acquired pneumonia are Enterobacteriaceae, *Staphylococcus aureus*, *Pseudomonas aeruginosa*, and *Acinetobacter baumannii*.^29^ In some cases, there may be multiple bacterial infections, concurrent bacterial pneumonia and influenza, or secondary bacterial infections after an exacerbation of a viral infection. The most common pathogen for secondary bacterial infections is *Staphylococcus aureus*, followed by *Streptococcus pneumonia*, *Haemophilus influenza*, and group A streptococcus.^30^ Both lnc‐ANRIL and miR‐125a are associated with the severity and pro‐infammatory factors level of sepsis, also are high value predictive biomaker for short‐term sepsis risk and 28‐day mortality.^31^ Additionally, lnc‐ANRIL/miR‐125a axis shows more efficient in the prediction of the sepsis risk, correlation with the organ damage and a series of inflammatory factors of sepsis.^32^ Overexpression of lnc NKILA leads to the upregulation of miR‐21, inhibition of JNK/NF‐κB pathway, and reduces immune response and lung fibroblast apoptosis and cell viability in pediatric pneumonia.^33^ lncRNA and miRNA are active regulator of both pro‐inflammatory and anti‐inflammatory responses, participating in the maintaince of a balance between pathogen clearance and normal morphology in the pulmonary system.

#### 3.1.2 Viral infections

Viruses cause almost a third of community‐acquired pneumonia, inculding respiratory syncytial virus (RSV), rhinovirus, influenza viruses, and coronaviruses.^34^ Viral‐bacterial co‐infection are common in children, about 25% of patients develop secondary bacterial infections after influenza A (H1N1) infection.^35^

Influenza A virus infection stimulates IFN‐β transcription, which in turn upregulates lnc‐ISG20 expression. lnc‐ISG20 acts as a ceRNA competing with ISG20 to bind miR‐326, reducing the inhibition of ISG20 translation and negatively regulating the replication of influenza A.^36^ There are also retroviruses known as prototype foamy virus (PFV) that cause no clinical symptoms after infection. Lnc‐RP5 binding to miR‐129‐5p and boost its expression as a result of the infection, repressing Notch1 to increase the unique internal promoter of PFV, thus active the expression of the viral transcriptional transactivator, be critical to the replication, expression, and transportation of virus.^37^

Since Decemeber 2019, the rapid spread of the novel severe acute respiratory syndrome coronavirus 2 (SARS‐CoV‐2) transmitted via respiratory droplets has led to a worldwide pandemic of the coronavirus disease 2019 (COVID‐19).^38^ Patients who are infected may show no symptoms for a brief period of time, and rapidly developing a high fever and severe respiratory symptoms (cough, shortness of breath, etc.), along with other nonspecific symptoms including malaise, myalgia, nausea, and vomiting.^39^

It has been found that miR‐146a‐5p, miR‐21‐5p, and miR‐142‐3p are relatively lowly expressed and miR‐15b‐5p is highly expressed in the serum of COVID‐19 patients. Metastasis‐associated lung adenocarcinoma transcript 1 (MALAT1) can bind to miR‐146a‐5p and miR‐142‐3p and shows a negative correlation with both.^40^ Furthermore, patients with lower miR‐146a‐5p expression levels responded to Tocilizumab less well. MALAT1 and nuclear‐enriched autosomal transcript 1 (NEAT1) expression was elevated in SARS‐CoV‐2‐infected bronchial epithelial cells.^41^

NEAT1 target miR‐21 in allergic rhinitis to inhibit its anti‐inflammatory effects and exacerbate the extent of the allergic inflammatory response.^42^ Therefore, it is hypothesized that MALAT1 silencing miR‐146a‐5 and NEAT1 targeting miR‐21 may serve as targets for COVID‐19 therapeutic targeting.

### 3.2 ARDS

ARDS was known as noncardiogenic respiratory failure with severely impaired pulmonary function, hypoxemia, and decreased pulmonary compliance, with around 30 million patients every year^43^ and mortality rate ranging from 34.9% to 46.1% depending on the severity.^44^ ARDS is caused by pulmonary infections (bacterial, viral, etc.), other significant infections (skin, genitourinary system, etc.), burns, particularly smoke inhalation, and all kinds of traumas.^45^ There are no specialized treatment options, and therapy is still reliant on lung‐protective mechanical ventilation.

When microorganisms, irritant mediators, and so on infiltrate the alveolar barrier, macrophages polarize from resident alveolar macrophages to the M1 phenotype at an early stage in response to Toll‐like receptors (TLR) induced by infection, releasing pro‐inflammatory factors such as IL‐1, IL‐6, and TNF‐α, which are the first hurdle of lung immune response,^46^ lncRNAs and miRNAs are early actived in this step, a large number of miRNAs, such as miR‐146, miR‐155, miR‐221, and miR‐222, have been noted to be stimulated upon TLR signaling activation, and lncRNAs such as Mirt2, THRIL, MALAT1, and lincRNA‐21 are also altered upon TLR activation and negatively regulate TLR signaling as well as suppress pro‐inflammatory factor expression.^47^ Recent studies have shown that the expression of miRNA could be potential biomarkers to determine the diagnosis of ARDS, suggesting that miR‐155, miR‐223, miR‐146, miR‐27a, and miR‐27b are upregulation, while miR‐150 is downregulated in ARDS patients.^48^ MALAT1 is highly expressed in inflammation‐activated macrophages, interacts with NF‐κB, inhibits TLR signaling, and lowers TNF‐α, IL‐6 and other inflammatory factors,^49^ MALAT1/miR‐146a axis is contributed to the stimulation of inflammatory response in ALI.^50^

while pro‐inflammatory Inflammation is exacerbated by further activation and release of pro‐inflammatory factors, chemokines, adhesion molecules, and so on. The main mechanism of ARDS is assumed to be the acute, widespread lung inflammation caused by this overwhelming immune response.^51^ HOTAIR affects the miR‐30a‐5p/PDE7A axis in LPS‐induced ARDS, increases the release of inflammatory factors, and exacerbates the pulmonary inflammatory response.^52^ LncRNA OIP5‐AS1 aggravates ALI/ARDS via the miR‐223/NLRP3 axis. Mechanically, lncRNA OIP5‐AS1 serve as miR‐223 decoys, preventing miR‐223 from binding with its direct target, NLRP3, and lowering pro‐inflammatory cytokines release and endothelial cell pyroptosis.^53^

## 4 CHRONIC LUNG INFLAMMATORY DISEASES

### 4.1 COPD

Airflow limitation with persistent respiratory symptoms and lung chronic inflammation are the main features of COPD, which is characterized by typical, prolonged dyspnea, cough and sputum, mainly due to long‐term immune response brought on smoking, occupational exposure, and so forth.^54^

Inducing oxidative stress from cigarette smoking results in severe cellular damage and an inflammatory response, which is a key pathogenic aspect of COPD. When cigarette smoke is applied to cells in vitro, it can cause cytotoxicity and an immunological response. MEG3 is a lncRNA highly expressed in bronchial epithelial cells which is regulated by the smoke extraction, sponge binding to miR‐181a‐2‐3, leading to higher cell apoptosis and inflammation.^55^ Since lnc RP11‐86H7.1 interacted with miR‐9‐5p through a ceRNA mechanism, which would lower miR‐9‐suppression 5p's of NFKB1 production in bronchial epithelial cells, Zhao and colleagues hypothesized that such ternary network may boost PM2.5 related COPD.^56^

Increasing studies have shown that lncRNAs and miRNAs may be closely related to the occurrence and development of COPD, could be potential biomarkers and therapeutics.^57^ As a predictor of susceptibility, NEAT1 is also associated with disease severity and inflammation level, has increased expression in the peripheral blood of COPD patients, and functions in this way by down‐regulation the expression of miR‐193a.^58^

As a therapeutic way, RNA drugs are highly specific and safe, may be one of the ideal method of administration for COPD.^54^ miR‐146 affects pulmonary bronchial epithelial cells to have anti‐inflammatory effects in a number of chronic lung disorders. Since lnc‐PVT1 affects miR‐146, its expression level can be utilized to distinguish acute exacerbation of COPD (AECOPD) patients and stable cope patients. lnc‐PVT1 expression also predicting COPD susceptibility and AECOPD risk, and is positively correlated with inflammation factors and disease severity stages.^59^ Through RNA sequencing and Bioinformatics prediction, Qian and colleagues created a miRNA‐mRN‐lncRNA ternary interaction network in nonsmoking COPD patients and projected that miR‐218‐5p/miR15a‐RORA‐LOC101928100/LINC00861 and miR‐218‐5p/miR15a‐TGF3‐RORA‐AS1 interactions play a significant role in the pathogenesis of nonsmoking COPD patients.^60^

### 4.2 Asthma

Asthma is one of the most prevalent chronic inflammatory diseases, typically characterized by airway hyperresponsiveness and airway obstruction, and nonspecific airway symptoms caused by specific triggers (such as allergens, environmental factors, infections, etc.). Airway remodeling, including thickening of the airway wall and narrowing of the airway, can occur in younger children as a result of epithelial injury, cilia failure, cupular cell proliferation, fibroblasts, and growth of airway smooth muscle cells.^61^ For now, there are approximately 300 million asthma patients worldwide.^62^ Over the last 40 years, there has been a significant surge on the prevalence, morbidity, and mortality associated with asthma among children.^63^

Through its association with miR‐124, lnc‐NEAT1 triggers the release of a number of inflammatory cytokines, and it is linked to a high risk of severe asthma exacerbations.^64^ Besides this, lnc PVT1 suppresses the expression of miR‐149, increases inflammation in small airway epithelial cells, and impairs cellular defense barrier function.^65^ Asthma‐related lung inflammation is mediated by CD4+ T cells, and asthma development is facilitated by enhanced T cell differentiation of Th2 cells. MiR‐155 is one of the most important regulators in immune system, could be considered as a potential novel target for asthma as its indispensable contribution to Th2‐related inflammation.^66^ The ongoing Phase II clinical trial in mycosis fungoides patients is currently evaluating Cobomarsen, an Oligonucleotide Inhibitor of miR‐155, aims to determine the safety, tolerability, and pharmacokinetics (NCT03713320).^67^

Airway smooth muscle cells (ASM) are the primary effector cells in asthma, and several studies have revealed that ASM cells proliferate in asthma patients and promote a more contractile ASM phenotype in response to inflammatory factor stimulation.^68^ The upstream lnc NEAT1 was identified to modulate SLC26A2 expression by targeting miR‐9‐5p, enhance ASM cell proliferation, migration, contraction, and boost inflammation in child asthma patients.^69^

Platelet‐derived growth factor BB (PDGF‐BB) stimulates Malat1 expression in airway smooth muscle cells, Malat1 interacts with miR‐150 by a ceRNA mechanism, significantly enhances the essential translation initiation factor, eIF4E, and Akt signaling, promotes airway smooth muscle cells proliferation and migration, airway remodeling in asthma.^70^ Meanwhile, there are studies demonstrated that lnc GAS5 acts on miR‐10a/BDNF axis,^71^ lnc PVT1 acts on miR‐590‐5p/FSTL1 axis,^72^ lnc Malat1 acts on miR‐150‐eIF4E/AKT axis^70^ influencing ASM cell proliferation and migration in asthma.

### 4.3 Pulmonary fibrosis

Idiopathic pulmonary fibrosis (IPF) is a chronic, progressive interstitial lung disease mainly occurs in the elderly. It has a poor prognosis, a high mortality rate, and prone to acute exacerbation to respiratory failure.^73^ Its etiology is still unknown, however, is frequently associated with smoking, occupational exposure, air pollution, and infection. Damage and repair of alveolar epithelial cells owing to numerous causes, activation of fibroblasts, stimulation of fibropathic proliferation, recruitment and proliferation of immune cells such as alveolar macrophages and lymphocytes, and modulation of the fibrotic response.^74^ This gradual destruction of the normal lung structure results in progressive decompensation of lung function, and once IPF initiates, it is irreversible. Although there are medications available to slow its progression, they cannot halt the disease's advance.^75^

The epithelial‐mesenchymal transition (EMT) is critical in the advancement of pulmonary fibrosis since epithelial cells able to de‐differentiate then differentiate into mesenchymal cells, which continually produce and accumulate extracellular matrix, directly tied to signaling pathways such as TGF‐1, Smad, and ERK/MAP.^76^ Silicosis is caused by long‐term silica inhalation and deposition in the lungs, which leads to diffuse pulmonary fibrosis. Macrophages are prompted to release TGF‐1, which causes lnc ATB expression in epithelial cells and binds to miR‐200c to increase ZEB1 expression in silica‐induced silicosis pulmonary fibrosis.^77^ In addition, miR‐29b‐2‐5p and miR‐34c‐3p are targets for sponging binding downstream of lnc ATB, upregulating the expression of MEKK2 and NOTCH2, enabling lnc ATB to contribute to the acceleration of the EMT process.^78^ Lung epithelial cells express more proliferation and EMT‐related genes when lnc NEAT1 interacts with miR‐29c.^79^ lnc RFAL binds to miR‐18a and activates fibroblasts via CTGF to accelerate the process of lung fibrosis,^80^ lnc RFAL also suppresses miR‐26a expression, therefore inhibiting miR‐26a's anti‐fibrotic action. A mutually inhibitory feedback loop established between miR‐26a and Smad2, leads lnc PFRL to increase fibroblast proliferation and transform into myofibroblast.^81^

In a study by Liu and colleagues in the mice silica‐induced lung fibrosis model, the overexpression of lnc PCAT29 elevated miR‐221 expression, inhibited TGF‐β1 in lung fibroblasts, and slowed the lung fibrosis process through the RASAL1/ERK1/2 signaling pathway.^82^ Through the miR‐326/SP1 axis, lnc SNHG1 enhances fibroblast migration, invasion, and fibrogenic molecule production,^83^ whereas lnc SHNG6 promotes fibroblast activation and collagen accumulation via the miR‐26a‐5p/TGF‐1‐smads axis, inducing lung fibrosis in mice.^84^

## 5 LUNG CANCER

Lung cancer is the leading cause of cancer‐related deaths, nonsmall cell lung cancer (NSCLC) which has an aggressive clinical course, is the most common type of lung cancer, adenocarcinoma is the most common histologic subtype of NSCLC.^85^, ^86^ There is mounting studies suggest that inflammation involved in the every step of lung cancer, including malignant transformation, tumor invasion, and metastasis.^87^ Besides, immune system also struggling with the cancer immunoediting which leading to the cancer elimaination, equilibrium, and escape.^88^ The tumor microenvironment (TME) comprises immune cells that interact with malignant cells, supporting tumorigenesis and invasion. Cell‐based immunotherapy targeting immune cells to promote antigens generation in T‐cells,^89^ or to help identify tumor antigens in DC cells.^90^ NcRNAs, has been investigated by many studies, play the role as regulators or hallmarks in cancer, and involved in cancer immunoediting process, may be an ideal target for therapies.^91^

### 5.1 lnc‐miRNA acts in immune checkpoints

Immune checkpoints molecules are crucial for modulating immune responses. Typically, cytotoxic T‐lymphocyte‐associated antigen 4(CTLA4) and Programmed death‐ligand 1 (PD‐L1), mainly expressed on the surface of T cell surface, induce T cells initial respone to antigen or limit the activity of T cells to respone to inflammation. In the tumor microvironment, tumor cells may inhibit the T cell responses by secret the immunosuppressive proteins that bind with the CTLA‐4 and PD‐1, shows to be a central target of immunosuppression in the therapeutic antitumor immunity.^92^ As a phase III clinical trial in extensive‐stage SCLC patients reported that first‐line treatment plus immunotherapy, which targeting the PD‐1 and PD‐L1 pathway, significantly prolong overall survival.^93^

nuclear‐enriched autosomal transcript1 (NEAT1) is overexpressed in lung cancer, and could be regarded as an oncogene to enchance the poliferation and antiapoptosis of cancer cell,^94^ is related to induce the immune tolerance of DCs and T‐cell responses, by targeting miR‐3076‐3p to regulating the expression of NLRP3 inflammasome.^95^ Via the miR‐155/Tim‐3 pathway, repression of NEAT1 inhibited CD8+ T cell apoptosis and raised the cytolysis activity.^96^ LINC01140 is increased in lung cancer tissues, leading to the reduction of miR‐377‐3p and miR‐155‐5p. This upregulation results in a increase in PD‐L1 and c‐Myc, both recognized as cancer‐promoting factors. The heightened expression of LINC01140 associated with poor survival and immune escape of lung cancer cells.^97^ The blockade of immune checkpoints has shown efficacy in activating exhausted T‐cells.^98^ Overexpression or inhibition of ncRNAs could be developed as a method to improve anticancer immune responses.

### 5.2 lnc‐miRNA in tumorigenesis and invasion

A collective lncRNAs exhibited aberrant expression levels in tumors when compared to their paired normal tissues, such as MALAT1, H19, and MEG3, displayed differential expression across all stages of lung cancer formation. MALAT1, one of the earliest discovered genes linked to lung cancer, is seen as a predictive indicator for lung cancer. It is primarily located in nuclear speckles and interacts with numerous transcription factors, chromatin modifiers, and RNA binding proteins (RBPs) to regulate gene expression at both the transcriptional and post‐transcriptional levels.^99^ Despite not being required for normal tissue growth and development, MALAT1, act as an essential lncRNA that contributes to tumor growth, invasion, and metastasis.^100^ METTL3 enhances the stability of MALAT1 and modifies it via m6A methylation, which facilitates MALAT1's spongy binding to miR‐1914‐3p. This, in turn, boosts YAP expression and triggers YAP‐induced metastasis and invasion in NSCLC.^101^

HOTAIR is linked to the formation of several tumors, and one of the mechanisms is that HOTAIR controls several downstream targets via different signaling pathways, which are linked to tumor cell motility, proliferation, angiogenesis, invasion, and drug resistance.^102^ The potential mechandisms have been identified wherein HOTAIR mediates either miR‐613^103^ or the miR‐217/DACH1 signaling pathway,^104^ to facilitating the invasion and metastasis of NSCLC. In NSCLC cells, miR‐221 reduces HOTAIR expression and increases apoptosis.^105^ By interacting with miR‐149‐5p, HOTAIR drives the emergence of cisplatin resistance in NSCLC.^106^ Other lncRNA‐miRNA interactions have also been demonstrated to contribute to lung tumorigenesis. For instance, in a feedback loop formed by lnc ZEB1‐AS1 and miR‐409‐3p/ZEB1, miR‐409‐3p functions as a regulatory bridge, is suppressed by lnc ZEB1‐AS1 thereby upregulating ZEB1, which then binds to the lnc ZEB1‐AS1 promoter regions to promote tumorigenesis.^107^ Lnc GACAT1 expression is increased in NSCLC tissues and cell lines, spongiosely binds miR‐422a and inactivates the YY1 transcription factor (YY1), which may be associated with poorer clinical outcomes for patients.^108^

## 6 ncRNAs IN CLINICAL USE

RNA‐based therapies, which mainly include antisense oligonucleotides (ASOs), smll interfering RNAs(siRNAs), short hairpin RNAs (shRNAs), CRISPR–Cas9, miRNA mimics, are designed to regulate RNA expression in vitro and in vivo. 11 such therapeutics have received approval from the FDA or the European Medicines Agency.^109^ However, the clinical application of ncRNA‐based therapies presents cannot ignore the challenges like instability of single‐stranded RNAs in vivo, cell permeability and metabolism by organs such as the liver or kidney.^110^

Preclinical studies on ncRNA in mouse models have primarily focused on chemical modifications or delivery methods involving lipid nanoparticles, viral vectors, and bacteriophages. Lipid nanoparticles, spherical assemblies that assembled with ncRNAs, can deliver these molecules to specific body regions, particularly overcoming barriers in the airway surface associated with alveolar macrophages.^111^ Inhalation of drugs delivered via lipid nanoparticles to the lungs can prolong release and increase bioavailability.^112^

In addition to their therapeutic potential, ncRNAs can serve as diagnostic biomarkers or indicators of therapy resistance and prognosis in lung cancer. Noninvasive methods such as liquid biopsy can detect ncRNAs released from tumors into peripheral fluids like blood and urine, providing an easily accessible and less harmful testing method.^113^ Targeting to the tumor‐infiltrating lymphocytes (TILs), tumor‐associated macrophages (TAMs), and other immune cells also offers a new sight for cancer biomarkers.^114^ Whereas, larger patient cohorts are needed to predicat and validate the use of ncRNA‐based biomarkers in lung cancer.

## 7 DISCUSSION AND PROSPECT

Lung diseases often manifest in combination with other systemic diseases or present as a simultaneous occurrence of multiple lung disorders. Microorganisms, for example, can initiate pneumonia at an early stage, but severe pneumonia may subsequently tigger ARDS or even MODS. In a long term, pulmonary fibrosis is one of the most common combinations. Notably, pulmonary fibrosis increases the risk of developing lung cancer by 7%–20%.^115^

LncRNAs and miRNAs play integral roles in various pulmonary inflammatory diseases, exhibiting distinct patterns of expression, diverse combinations, and differential functions. Several star lncRNAs have been throughly investigated across a range of diseases. Among these, MALAT1 and NEAT1, are implicates in most of both acute and chronic pulmonary inflammation diseases. MALAT1 and HOTAIR are found expression in various tumors, including lung cancer. Besides, we observed there is a surge in advanced research on the ceRNA mechanism in the analysis of interactions between lncRNA and miRNA. In this mechanism, lncRNA act as miRNA decoys to prevent miRNAs from binding with their target mRNA. We assumed that this may be the reason rapidly developed bioinformatic and computational biology provide the tools to predicate RNA targets and analyze specific RNA regulation. Even though raising different voices that ceRNA only performs a slight function in miRNA regulation, it is unlikely that a single ceRNA to have any biologically significant effects on the expression of genes or the activity of miRNAs.^11^, ^116^ Most of RBP and miRNAs regulate gene expression posttranscriptional have thousands of binding sites, the numbers of these binding sites are highly dynamic as transcription renders, only strong binding sites are likely to be altered by crosstalk effects.^117^

As lung constant exposure to the external through respiration, it is more vulnerable to infection than other systems, and inflammation represents in many lung diseases. Ongoing research on lncRNA‐miRNA interaction in pulmonary inflammatory diseases shows their key regulatory and potential as biomarkers. However, the translation of all the findings into clinical application as biomarkers for diagnosis or therapeutic targets necessitates a comprehensive understanding of the underlying curative mechanisms, the roles and processes of which are yet unclear.

## AUTHOR CONTRIBUTIONS

**Jiaqi Li**: Visualization; writing—original draft. **Shengyu Huang**: Resources; writing—review & editing. **Liangliang Shi**: Validation. **Guochang Chen**: Writing—review & editing. **Mingzhuo Liu**: Supervision. **Guanghua Guo**: Supervision.
